# The importance of accounting for larval detectability in mosquito habitat-association studies

**DOI:** 10.1186/s12936-016-1308-4

**Published:** 2016-05-04

**Authors:** Matthew Low, Admasu Tassew Tsegaye, Rickard Ignell, Sharon Hill, Rasmus Elleby, Vilhelm Feltelius, Richard Hopkins

**Affiliations:** Department of Ecology, Swedish University of Agricultural Sciences, Box 7044, 75007 Uppsala, Sweden; Ethiopian Institute of Water Resources, Addis Ababa University, Addis Ababa, Ethiopia; Unit of Chemical Ecology, Department of Plant Protection Biology, Swedish University of Agricultural Sciences, Box 102, 23053 Alnarp, Sweden; Department of Earth Sciences, Uppsala University, Uppsala, Sweden; Natural Resources Institute, University of Greenwich, London, UK

**Keywords:** *Anopheles arabiensis*, *Anopheles gambiae* complex, *Aedes*, *Culex*, Malaria, Presence, Abundance, Bayesian hierarchical modelling, WAIC

## Abstract

**Background:**

Mosquito habitat-association studies are an important basis for disease control programmes and/or vector distribution models. However, studies do not explicitly account for incomplete detection during larval presence and abundance surveys, with potential for significant biases because of environmental influences on larval behaviour and sampling efficiency.

**Methods:**

Data were used from a dip-sampling study for *Anopheles* larvae in Ethiopia to evaluate the effect of six factors previously associated with larval sampling (riparian vegetation, direct sunshine, algae, water depth, pH and temperature) on larval presence and detectability. Comparisons were made between: (i) a presence-absence logistic regression where samples were pooled at the site level and detectability ignored, (ii) a success *versus* trials binomial model, and (iii) a presence-detection mixture model that separately estimated presence and detection, and fitted different explanatory variables to these estimations.

**Results:**

Riparian vegetation was consistently highlighted as important, strongly suggesting it explains larval presence (−). However, depending on how larval detectability was estimated, the other factors showed large variations in their statistical importance. The presence-detection mixture model provided strong evidence that larval detectability was influenced by sunshine and water temperature (+), with weaker evidence for algae (+) and water depth (−). For larval presence, there was also some evidence that water depth (−) and pH (+) influenced site occupation. The number of dip-samples needed to determine if larvae were likely present at a site was condition dependent: with sunshine and warm water requiring only two dips, while cooler water and cloud cover required 11.

**Conclusions:**

Environmental factors influence true larval presence and larval detectability differentially when sampling in field conditions. Researchers need to be more aware of the limitations and possible biases in different analytical approaches used to associate larval presence or abundance with local environmental conditions. These effects can be disentangled using data that are routinely collected (i.e., multiple dip samples at each site) by employing a modelling approach that separates presence from detectability.

**Electronic supplementary material:**

The online version of this article (doi:10.1186/s12936-016-1308-4) contains supplementary material, which is available to authorized users.

## Background

Accurately estimating site-specific mosquito larval abundance is central to vector control strategies for identifying breeding habitat characteristics [1, 2], determining the need for control and/or evaluating the efficacy of mosquito control programmes [3–5]. Habitat association studies have linked *Anopheles* mosquito larval presence or abundance to a suite of environmental factors related to the water body, such as depth [6–8], temperature [7, 9], algae [8, 10, 11], riparian vegetation and shading [1, 5, 10]. Despite this there is still uncertainty regarding the importance of some factors because of the between-study variation in these patterns. Although these differences may be partly explained by selective, cross-sectional sampling [6] or species-specific preferences [2], one possibility that has received relatively little attention is the behaviour of the larvae under different environmental conditions. There is increasing evidence that mosquito larvae adjust their behaviour in response to surface disturbances or predation risk [12, 13], temperature [14] and water nutrient levels [14, 15]. Yet, how these and other factors translate into the probability of larvae being sampled, and the subsequent impact on the results of habitat-association studies, has never been explored.

There is a need to incorporate more relevant biological detail into our modelling of malarial mosquito ecology [16]. One relatively simple way of doing this with regards to larval-habitat associations is to use a framework that indirectly includes environmental effects on larval behaviour: i.e., allows for detection probability to vary with environmental variables. This can be done by extending the general linear model to include a detection parameter calculated from sampling each site multiple times [17, 18]. Mosquito larval studies are unusual in that they use a data collection method that can be used to calculate the probability of detection without additional sampling effort. Larvae are typically collected using dip sampling, in which multiple samples are collected from each site using a dipper; these are usually combined to give site-level estimates of presence or density [19]. A simple modification to this method is to separately record the results for each dip sample rather than pooling them [e.g., 3]; this repeated sampling at each site enables detection probability to be estimated. Thus, instead of directly relating all environmental factors of interest to larval presence, environmental variables can be modelled as influencing presence and/or detection probability. Such models would therefore not only improve the accuracy of presence estimates by accounting for imperfect detection, they would also make more biological sense in that environmental factors that influence larval detectability would not be erroneously linked to predicting mosquito presence.

In this study, data were used from a dip sampling survey in Ethiopia to examine if observations were confounded by imperfect detection, and determine if different environmental variables influenced larval presence compared to detection probability. First, as a comparison to previous studies, the data were analysed using the most common approach [19]: i.e., aggregating the site data response variable into a single presence/absence value. Second, a less common approach was used that accounts for how many times each site was sampled and how many of these samples contained larvae (binomial distribution: successes per number of trials). There was an expectation of differences between the results of the first and second analyses because the first models only the larval presence and ignores detectability, while the second incorporates some measure of detectability, although this is confounded with presence. Third, a mixture model was developed that separately estimates presence and detection to allow detectability to be explicitly disentangled from presence/absence. This allowed an examination of whether a more complex modelling approach had greater support and predictive capability over simpler methods. In addition, the mixture model also allowed a combination of different environmental variables within the model’s separate presence and detection components to see if there was support for some variables being more important for detection, and some more important for larval presence. Finally, the effects of environmental variables on presence and detection were modelled to estimate how variation in these factors influenced presence and how many dip samples were required to confidently state whether a site contained larvae.

## Methods

### Study area and larval sampling

Twenty-six sampling sites in both artificial and natural water habitats were chosen in farming and grazing lands near to lake Abaya and Chamo in Ethiopia [Additional file 1; ~1100 m above sea level (a.s.l.)] where malaria transmission is common. Sites were sampled 5–10 times on each sampling occasion using a 300 ml dipper, with each site being visited twice, 10–14 days apart. The time between visits meant that any larvae present at the first sampling should have emerged before the second visit. Dip samples were taken at random locations across the body of water, with the exception of river sites where flowing areas were excluded. The total number of site visits used in analyses was 44, since eight sites became flooded with moving water between the first and second visit.

Larvae were identified in the dipper at the time of collection as *Anopheles* (total n = 569) or non-*Anopheles* (n = 676). *Anopheles* larvae were stored in 78 % ethanol for later morphological confirmation and showed that misclassification in the field was extremely low (~0.3 %). Of the *Anopheles*, 118 could be further classified, with 117 being from the *Anopheles gambiae* sensu lato species complex and one as *Anopheles garnhami*. There was large within-site variation in the number of larvae sampled during dipping, with 14 of the 23 sites where *Anopheles* larvae were found recording at least one dip during sampling that contained no *Anopheles* larvae (proportion of dips with no larvae at sites where *Anopheles* were known to be present: mean ± SD = 0.247 ± 0.255; range 0–0.8).

### Selection and measurement of environmental factors

Site factors were measured at each visit that included water and local environmental variables previously linked to the presence or abundance of *Anopheles* larvae: i.e., water pH, depth and temperature, as well as the presence of algae, amount of riparian vegetation and sun/shading on the water (see references above). Although other factors likely regulate *Anopheles* presence, the study specifically focussed on these variables for three reasons: (1) the aim was not to exhaustively determine the variables linked to *Anopheles* presence, but rather to demonstrate how different statistical models can change the interpretation of the importance of different variables, (2) to show how presence and detectability can be confounded, factors were used that varied in their probability of being related to these two processes (e.g., riparian vegetation [presence], if it was sunny at the time of collection [detection], and water depth [presence + detection]), and (3) by limiting the search to factors that have been previously identified in at least one study as being potentially important, this avoided many of the problems associated with the exponential growth of the number of possible models during model selection [20].

For water variables, a portable water chemistry meter (HI-991301, Hanna Instruments, USA) was used to record water pH (±0.01) and water temperature (±0.5 °C) at each site following the manufacturer’s instructions. In addition, water depth (±0.5 cm) at the location of each dip sample was measured. Dip sampling was restricted to areas within sites with water depths of <200 mm in slow flowing or still water because this range of water depths may influence egg laying (shallower water favours *Anopheles* larval survival; [21]) in addition to sampling efficiency. For environmental parameters, measurements were taken of the proportion of riparian vegetation around the water body that was >30 cm high (including tall grass, crops and trees), whether it was a sunny or cloudy day and whether there was visible algal growth in the water body.

### Statistical modelling

Three modelling approaches were compared. The first two model forms represent methods that have been previously used in larval habitat-association studies [19], with the third being an extension of these models to separate the effects of presence from detectability [18]. The first model form (‘presence–absence’) is a generalized linear (mixed) model (GLMM) where the response variable of larval presence/absence is based on aggregating all dip samples to a single site value of present (1) or not (0). For this, a single sample binomial (Bernoulli) distribution is used with a logit-link (a classic logistic regression), to calculate the probability of a site containing *Anopheles* based on particular explanatory variables. This method assumes that if larvae are present you will find them at least once during sampling, and thus does not account for detectability. The second model form (‘success-trial’) is also a logit-link binomial GLMM, but it incorporates information about the number of samples taken at each site and the number of dips in which larvae were found at each site; i.e., the response variable for each site contains two pieces of information, the number of dips taken (‘trials’) and the number of dips that contained larvae (‘successes’). Unlike the first model form, this allows for a range of probabilities in the response variable from 0 to 1. One consequence of this when examining larval-habitat associations is that the model will report relationships between not only factors related to larval presence, but also to the number of dip samples at each site containing larvae. Thus, the proportion of samples containing larvae at each site will be a function of factors related to presence/absence and detectability or abundance. Although results from such analyses may include explanatory variables that are related to detectability, they are unable to separate which factors are correlated to presence and/or detectability.

The third model form (presence-detection mixture) explicitly separates factors relating to presence at a site from those of detectability. This essentially involves combining the first two model forms in a way that disentangles these key elements that determine whether larvae are found when sampling a water body: the probability of observing larvae in a scoop becomes the product of true presence (i.e., larvae are present or not) × detection probability (i.e., the probability of sampling larvae when they are present). This means that by explicitly estimating detection probability based on the multiple samples taken at each site, the true presence can be derived from the field observations (Additional file 2). More importantly for habitat-association studies, different variables can be assigned to detection or presence, allowing the effect of explanatory variables on presence or detection to be disentangled. The detection component of the model was based on the repeated samplings at each site during each visit. These were modelled for each dip as a Bernoulli distribution (larvae present or not in an individual dip), with site included as a random effect to account for the repeated samplings. From this base model, variables explaining detection probability could be added to see what factors were linked to underestimation bias during sampling. This is then combined with explanatory variables in the presence part of the model to see which factors were important in explaining presence/absence of *Anopheles* larvae once detection was accounted for.

For the first two model forms (presence–absence and success-trial GLMM), the effect size of explanatory variables of interest were examined by using two commonly used approaches for model selection: multi-model inference using the likelihood framework [20] and stepwise backwards selection using p values. In all cases, the analysis started with the full model including all explanatory variables as fixed effects and site location as a random effect, implemented using the function ‘glmer’ (from the ‘lme4’ package; [22]) in R [23]. For multi-model inference, the balanced set of candidate models containing all possible combinations of fixed factors (without interactions) were generated using the package ‘MuMIn’ [24]. Models were ranked using the sample-size corrected Akaike information criterion (AICc), and from this AICc-based relative importance weights and model-averaged parameter estimates for each factor were generated. For stepwise backwards selection, the factor with the highest p value was eliminated in a stepwise process until AIC was minimized, and examined p values for the explanatory variables that remained in the final model.

Presence-detection models—the third model form—were implemented in a Bayesian hierarchical framework in JAGS (just another Gibb’s sampler; [25]) called from R using the ‘rjags’ package. This allowed us to combine individual-level scoop data within the detection model, with the site-level data for the presence model (model code is in Additional file 2). For formal model comparisons, the Watanabe-Akaike information criterion (WAIC) was used, as has been recommended for Bayesian mixture models [26], using the likelihood and log-likelihood of the model for each iteration of the MCMC chain. However, because the goal of this study was to demonstrate the importance of different variables in how they might influence larval presence versus detection, the model selection approach was similar to Hobbs et al. [27] in that inference was based on a full model structure based on biological knowledge of the system. Thus, included in the detection part of the model were four of the explanatory variables that could conceivably influence detection (sunshine, water depth, temperature and algae), and for presence, five variables (riparian vegetation and algae, as well as water pH, depth and temperature). From this, the overlap of the posterior distributions with 0 were examined for each coefficient. By doing this the probability of a coefficient being positive or negative can be directly calculated (with a probability of 50 % meaning the mean estimate for the coefficient = 0 and has no predictive value); thus coefficients that largely overlap zero can be considered unimportant to the process being modelled. For all Bayesian models vague priors were used, chains were run for 50,000 iterations with 10,000 burn-into allow stabilisation of the chains, and assessed convergence based on three MCMC chains using visual inspection and the Gelman and Rubin diagnostic [28]. For all models, continuous variables were mean-centred to improve convergence.

## Results

### Generalized linear models

There were clear differences between the predictions of the presence–absence and success-trial GLMMs that reflected the different information contained in their response variables. For the presence–absence model, there was strong evidence that riparian vegetation had a negative effect on larval presence (−) (Table 1). There was also moderate support for water depth (−) and pH (+) being related to larval presence if using multi-model inference (relative importance weight for both variables = 0.69, with both variables included in the highest-ranked model; Additional file 3). However, if stepwise backwards selection based on AIC was used for model selection, neither water depth nor pH was significant (p < 0.05) in the final model (although if either term was removed the remaining term was significant; see Table 1 for all parameter estimates and their associated uncertainties). For the success-trial model, in addition to riparian vegetation (−) there was strong evidence that water depth (−) and sunshine on the water surface (+) influenced the probability of finding larvae in a dip sample (Table 1; Additional file 3). This additional strength of support for effects of sunshine and water depth in the success-trial model suggests these variables were linked to the probability of finding larvae in an individual scoop sample (i.e., detectability), rather than to site presence.

**Table 1:** Mean estimates ± SE for coefficients in logit-link GLMMs where ‘presence–absence’ models the response at the site level, and ‘success-trial’ models the response as a binomial with information on the number of samples collected at each site

Parameter	Presence-absence GLMM				Success-trial GLMM			
	Multi-model inference		Backwards selection		Multi-model inference		Backwards selection	
	Estimate	RIW	Estimate	p value	Estimate	RIW	Estimate	p value
Intercept	−0.16 ± 0.6	–	0.25 ± 0.52	0.62	−4.6 ± 1.2	–	−4.38 ± 1.1	<0.001
Vegetation	−0.14 ± 0.07	1.0	−0.14 ± 0.06	0.03	−0.18 ± 0.04	1.0	−0.17 ± 0.04	<0.001
Depth	−0.29 ± 0.29	0.69	−0.37 ± 0.24	0.13	−0.52 ± 0.21	0.96	−0.60 ± 0.16	<0.001
pH	1.6 ± 1.7	0.69	1.77 ± 1.34	0.18	0.51 ± 0.85	0.40	–	
Sunshine	0.40 ± 0.71	0.26	–		3.9 ± 0.96	1.0	3.9 ± 0.93	<0.001
Temperature	0.03 ± 0.08	0.24	–		0.08 ± 0.1	0.43	–	
Algae	0.28 ± 0.84	0.31	–		0.62 ± 0.81	0.52	–	

Estimates are multi-model-averaged shrinkage estimates with variable relative importance weights ‘RIW’ and from stepwise backwards selection (estimates and p values from the final model). Vegetation = percentage of tall riparian vegetation, Depth = depth at each sampling point, Sunshine = sunny day with sun on the water surface, Temperature = water temperature, Algae = visible algal presence

### Presence-detection mixture model

There was overwhelming support for modelling separate presence and detection components in a mixture model compared to the equivalent success-trial GLMM (success-trial GLMM model versus mixture model WAIC = 199.2 versus 86.6 respectively). This was based on separating the three variables identified in the success-trial GLMM analyses (riparian vegetation + sunshine + depth) into the different components of the mixture model: presence (riparian vegetation) and detection (sunshine + depth).

**Fig. 1:**
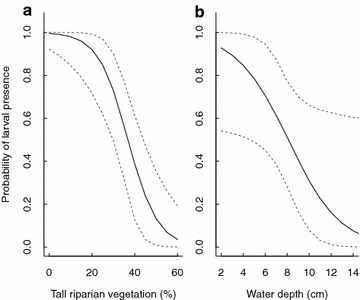
Estimated probability of larval presence as a function of **a** percentage of tall (>20 cm) riparian vegetation surrounding the water body and **b** mean water depth (cm) of the sampled water body. Means (*lines*) and 95 % credible intervals (*dashed lines*) were generated from the posterior distribution of the mixture model predictions (Table 2) when all variables except the one being modelled were held at their average value

**Fig. 2:**
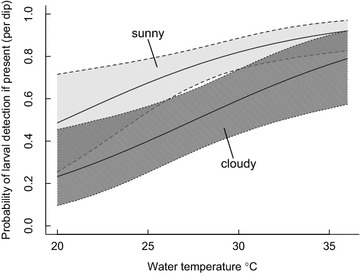
Estimated probability of finding at least one larva in a single dip sample taken from a site that contains mosquito larvae, relative to water temperature and sunshine on the water surface (sunny versus cloudy). Means (*lines*) and 95 % credible intervals (*dashed lines with shading*) were generated from the posterior distribution of the mixture model predictions (Table 2) when all variables except the ones being modelled were held at their average value

The results from the full model (presence = vegetation, depth, pH, algae and temperature; detection = sunshine, temperature, depth and algae) make it clear that sunshine + water temperature played a key role in detectability (Fig. 1), while riparian vegetation + water depth were strongly associated with larval site presence (>98 % of the posterior distribution range was above 0; Table 2; Fig. 2). There was weak to moderate support for algae and water depth influencing detectability, and water pH influencing site presence (85–95 % of the posterior distribution range >0; Table 2). If algae and water temperature were removed from the presence model because of their poor predictive effect, this strengthened the relationship between water pH and larval site presence (94 % probability of a + effect), and had some effect on the uncertainties of the other variables (Additional files 4, 5).

**Table 2:** Mean ± standard deviation of the posterior distribution (with 95 % credible intervals) for coefficients fitted in the full presence-detection mixture model

Parameter	Presence		Detection	
	Posterior distribution	Effect probability	Posterior distribution	Effect probability
Intercept	0.80 ± 1.51 (−1.0, 5.1)	–	−0.12 ± 0.34 (−0.80, 0.54)	–
Vegetation	−0.22 ± 0.14 (0.66, −0.08)	1	–	–
Depth	−0.76 ± 0.72 (−2.9, −0.03)	0.982	−0.06 ± 0.05 (−0.16, 0.04)	0.866
pH	1.46 ± 1.68 (−2.7, 4.3)	0.862	–	–
Sunshine	–	–	1.17 ± 0.35 (0.48, 1.86)	0.999
Temperature	−0.05 ± 0.29 (−0.85, 0.34)	0.518	0.18 ± 0.06 (0.06, 0.31)	0.999
Algae	0.45 ± 1.62 (−3.6, 3.1)	0.689	0.61 ± 0.41 (−0.19, 1.43)	0.934

For each coefficient the proportion of the posterior distribution that lies above (or below) zero is also shown as the ‘effect probability’: this is the probability that the effect of the parameter on larval presence or detection is in the direction specified by the sign in front of the coefficient (i.e., complete certainty = 1; complete uncertainty = 0.5). For example, there is a 98.2 % probability that water depth has a negative effect on larval presence and a 99.9 % probability that water temperature has a positive effect on detection. See Table 1 for definition of parameters. See Additional file 4: Table S4 for coefficient estimates and effect probabilities when terms with high overlap with zero are dropped from the model

**Fig. 3:**
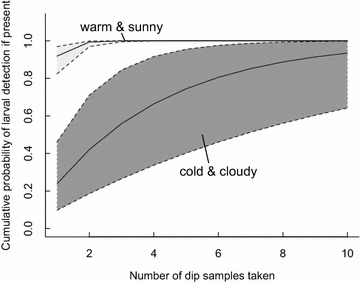
Estimated cumulative probability of finding larvae at a site based on the number of dip samples taken. Conditions are contrasted by warm water temperature (34 °C) + sunshine on the water (*light grey*) versus cool water (20 °C) + clouds (*dark grey shading*). Means (*lines*) and 95 % credible intervals (*dashed lines*) were generated from the posterior distribution of the mixture model predictions (Table 2) when all variables except the ones being modelled were held at their average value

Because detectability was strongly influenced by sunshine on the water surface and water temperature (Fig. 2; Table 2, Additional file 4), the number of dips required to confidently state whether larvae were present or not depended on the conditions at the time of sampling (Fig. 3) and how uncertainties were considered (Additional file 6). For example, on a sunny day with very warm water temperatures (~34 °C) there was >95 % certainty of a site’s occupancy based on only two dip samples. However, on a cloudy day in relatively cool water (~20 °C), you would need 11 dip samples to be >95 % certain of a site’s occupancy based on the mean detectability estimate. To have the lower range of the 95 % credible interval >0.95 certainty, then 30 dip samples were needed (Fig. 3; Additional file 6).

## Discussion

Mosquito habitat-association studies aim to identify factors linked to larval presence or abundance as the basis for control programmes or distribution models [2, 10]. However to ensure these studies are relevant, sampling protocols need to be designed and/or analysed in a way that the relationships between environmental factors and the probability of mosquito larval presence are not systematically biased. In this study, it was shown how the structure of the modelling framework can strongly influence relationships from data based on a common sampling protocol (i.e., fixed effort dip sampling [19]), particularly when detection probability is less than perfect. Accounting for imperfect detection is a major issue in ecological sampling studies (e.g., [29, 30]) yet detectability has not been accounted for in habitat-association studies of malarial mosquitoes (or indeed any mosquitoes; see [19]). The results show that a failure to consider detection probability and the factors that influence it have the potential to impact on results from presence-absence habitat-association models by: (1) underestimating the true occupancy of sites, (2) erroneously linking factors related to detection probability with those of larval presence, and (3) under- or overestimating the importance of factors related to larval presence.

There were some clear differences in the results from the three analytical approaches. The first approach, and the one most commonly used (presence-absence logistic regression [19]), assumes that detection at the site level in pooled samples is ~100 % and therefore any relationships between explanatory variables and larval presence are unbiased. This may be true if enough samples are taken at each site. However, the number of samples required to achieve this will depend on how easy it is to catch one larva: this will be related to the overall density and distribution of larvae in the water body [3, 31, 32] and larval behaviour (this study). When density is very low, the effort required to find larvae when present can be immense (e.g., >17,000 dips [3]). Thus, while rules of thumb on how many samples to collect at a site based on initial numbers of larvae caught may help reduce bias, there is no guarantee that bias will be eliminated unless detection probability is explicitly modelled. Despite this, comparing the results from the presence-absence model to the other models in this study shows that it was able to clearly identify the effect of riparian vegetation (important in all modelling frameworks), with effects of water depth and pH being much less certain.

The second approach (success-trial binomial) used the same data, but retained the success versus trials sampling information within the response variable. Here, the explanatory variable explains not only larval presence at the site, but also the proportion of dips at a site that contain larvae. So it makes sense that the results retain the main effect identified in the first analysis for larval presence (riparian vegetation) and include additional effects that are likely to explain the proportion of dips at a site that contain larvae (i.e., sunlight influences detection probability). This illustrates the importance of understanding the analytical method used when comparing habitat-association studies, as the results from these two methods contain different information despite using the same sample data. Interestingly between the first and second analysis, water depth went from being a factor with some support to a factor with strong support. This suggested that water depth operates on both presence and detection, with the success-trial analysis combining these effects. This interpretation is somewhat supported by the negative effects of depth on both presence and detection in the mixture model (Table 2). The variable of sunshine on the water at the time of collection was assumed a priori to be related only to detection; a comparison of the results of the presence-absence analysis (no effect) to the success-trial analysis (strong effect) shows this assumption is well supported.

The final modelling approach explicitly modelled presence and detection separately, in a way that allowed explanatory variables to influence these estimates. Here some of the patterns from previous analyses are repeated: i.e., riparian vegetation explaining larval presence and sunshine explaining detection probability. Water depth and pH were again highlighted as being of likely importance to larval site presence. Interestingly, water temperature was a very strong predictor of larval detectability, while in the previous analyses temperature showed no indication of being important. Table 2 suggests the reason for these seeming contradictory results; temperature has a negative effect on larval presence but a positive effect on detection. Thus, unless these processes of presence and detection are separately accounted for, the influences of different explanatory variables may be diluted or exaggerated.

Riparian vegetation had a clear negative relationship with larval presence (see also [5, 10, 33]). The mechanism driving this relationship is uncertain [2], but vegetation may negatively impact larvae directly or reduce egg laying through shading effects [5] or vegetative decay impacting on larval health [34]. Because riparian vegetation also includes farm crops (see also [1]), this effect could relate to pesticide and fertilizer use. Water depth beyond a few centimetres is known to reduce anopheline larval survival [21] because larvae will bottom-feed and deeper dives are energetically costly [35]. In this study, larval presence declined in a similar pattern; however, it is unknown whether lower larval presence/abundance in deeper water is a consequence of reduced egg laying or lower larval survival. Water pH is negatively correlated with larval survival and development [36]; however, in the range found in this study (pH 7–9) it would be unexpected for there to be strong effects. This suggests if the pH effect is real, it is most likely because pH was correlated with another measure not used in the analysis.

Many *Anopheles* species prefer sun-exposed and shallow water bodies, although whether this is because of direct effects on larval development or indirectly through habitat quality is unknown [2]. The results suggest that this relationship between sunlight and larval abundance may be more complex than previously acknowledged because sunlight and temperature appear to have a large effect on detectability. Because detection in water during warm sunny days versus cool cloudy days was compared, rather than sunny versus shaded sites [5], this provides confidence that differences in measured occupancy between these sites resulted from differences in detection rather than presence or abundance relating to the site itself. This expectation was verified in the analyses, with sunlight being an important component of the detection function. The same issue also relates to water temperature. Although there are food and temperature-related limits and constraints on mosquito larval development [37] that might be expected to influence egg laying, there are also temperature-related effects on larval behaviour [14, 15] that likely influence the probability of being sampled.

Factors highlighted as influencing detection almost certainly operate through their impact on larval behaviour. Conventional dipping methods sample near the water surface [19] and, thus, anything that changes the vertical distribution or aggregation of larvae can influence the probability of collection [32, 38]. For example, larvae forage more actively in cooler water, and hence are more likely to dive [15] and can stay down longer because of reduced metabolic oxygen consumption. This would increase their mixing in the water column and make it more difficult to sample when surface dipping. Likewise, sunshine warms the thin surface water layer (2 mm) where *Anopheles* live and often feed [2], meaning they would tend to remain in this narrow zone when it is sunny and retreat from it when the air temperature cools. Surface algae could influence behaviour by being an important larval food source [11, 39], since *A. gambiae* larvae are more likely to dive to the bottom for food under conditions of lower surface food availability [15]. In addition, the presence of algae likely supports a higher density of larvae, which increases the probability of sampling at least one larva per dip. Water depth will influence detection simply by providing a larger volume for larvae to distribute in, reducing the probability of being sampled.

## Conclusions

These results clearly show that detectability needs to be accounted for when undertaking mosquito larval surveys, especially if the density of larvae is low. Although the analyses focus on a presence–absence survey, detectability issues will also influence abundance estimates. Because environmental factors influence detection in different ways, and these factors are not uniform within the environment, systematic biases may emerge if they are not included when modelling habitat associations and species distributions. This has implications not only for studies of *A. gambiae*, but also between-species and between-life-stage comparisons [3, 4, 8] where factors are likely to influence focal species and life stages in different ways.

Attempts to deal with incomplete detection are partly incorporated into current sampling protocols, with multiple samples collected at each site. This approach will be reasonably effective if enough sampling at each site is undertaken. However when mosquito abundance falls to low levels, the amount of effort to confirm site occupancy increases exponentially [3]. Thus, accounting for detection uncertainty becomes vital in situations where larval abundance drops below a certain threshold. Identifying these thresholds and how they might vary under different environmental conditions is an important avenue of future studies. This is critical if particular combinations of environmental variables lead to expected low detection rates; in such cases the detection uncertainty and how it relates to environmental variables needs to be incorporated into the modelling framework. At the very least, researchers using dip sampling to analyse mosquito site occupancy must utilize the information from each individual sample to estimate detection probability in order to minimize biases in larval presence estimates.

## Additional files

10.1186/s12936-016-1308-4 Table of GPS co-ordinates and data for the 26 collection sites.
10.1186/s12936-016-1308-4 Bayesian BUGS model code for the presence-detection mixture model.
10.1186/s12936-016-1308-4 Table of outputs for the GLMM modelling using multi-model inference.
10.1186/s12936-016-1308-4 Table of coefficient estimates for the presence-detection mixture model when terms with effect probabilities of <90 % were removed.
10.1186/s12936-016-1308-4 Figure showing the relationship between pH and larval presence.
10.1186/s12936-016-1308-4 Table of cumulative probabilities of detecting larvae based on the number of dips, sunshine and water temperature.

## Abbreviations

GLMM: generalized linear mixed model
WAIC: Watanabe-Akaike information criterion
