# Supplementary material for: The importance of accounting for larval detectability in mosquito habitat-association studies
Source: Malar J. 2016 May 4;15:253. doi: 10.1186/s12936-016-1308-4 (PMC4855760; doi:10.1186/s12936-016-1308-4)
Supplement: Supplementary file 1 — 10.1186/s12936-016-1308-4 Table of GPS co-ordinates and data for the 26 collection sites. [file 12936_2016_1308_MOESM1_ESM.docx]

| Site | N | E | Altitude (m) | Type |
| --- | --- | --- | --- | --- |
| A | 06005.966' | 037034.692' | 1216 | Irrigation channel |
| B | 06002.578' | 037033.496' | 1223 | River fringe |
| C | 06009.799' | 037039.838' | 1186 | Irrigation channel |
| D | 06009.741' | 037039.989' | 1191 | Marsh |
| E | 06009.479' | 037039.821' | 1195 | Construction site |
| F | 06004.142' | 037035.288' | 1184 | River fringe |
| G | 06004.142' | 037035.124' | 1199 | River fringe |
| H | 06002.093' | 037034.193' | 1198 | Flood pool |
| I | 06003.903' | 037033.876' | 1211 | Construction site |
| J | 06009.644' | 037040.054' | 1179 | Irrigation channel |
| K | 06009.731' | 037040.208' | 1184 | Marsh |
| L | 06009.348' | 037039.868' | 1201 | Construction site |
| M | 06009.989' | 037040.066' | 1196 | Man-made pond |
| N | 06003.613' | 037036.114' | 1187 | Marsh |
| O | 05056.518' | 037032.048' | 1114 | Rain pool |
| P | 05055.836' | 037030.932' | 1127 | Artificial watering point |
| Q | 05054.067' | 037030.280' | 1117 | Flood pool |
| R | 05053.763' | 037029.897' | 1119 | Irrigation channel |
| S | 05052.856' | 037029.369' | 1120 | Irrigation channel |
| T | 05050.945' | 037028.073' | 1119 | Flood pool |
| U | 05056.535' | 037032.262' | 1123 | Artificial watering point |
| V | 05055.992' | 037032.081' | 1111 | Lakeside |
| W | 05051.124' | 037027.950' | 1134 | River fringe |
| X | 05051.212' | 037028.240' | 1135 | Irrigation channel |
| Y | 06003.433' | 037036.286' | 1183 | Natural pond |
| Z | 06004.340' | 037034.693' | 1204 | Marsh |

Additional Files for Low et al.

The importance of accounting for detectability in mosquito habitat-association studies – published in Malaria Journal

**Additional file 1: Table**

GPS co-ordinates and data for 26 collection sites used in this study
