# Supplementary material for: The importance of accounting for larval detectability in mosquito habitat-association studies
Source: Malar J. 2016 May 4;15:253. doi: 10.1186/s12936-016-1308-4 (PMC4855760; doi:10.1186/s12936-016-1308-4)
Supplement: Supplementary file 2 — 10.1186/s12936-016-1308-4 Bayesian BUGS model code for the presence-detection mixture model. [file 12936_2016_1308_MOESM2_ESM.docx]

**Additional file 2:**

Bayesian BUGS model code used for the presence-detection mixture model

## final model for Low et al. The importance of accounting for detectability in mosquito habitat-association studies

## BUGS-code implemented in JAGS

## code written by Matt Low

## mixture model with riparian vegetation and water pH for presence (site data)

## sunshine and water temperature for detection (individual scoop data)

model{

#priors

#presence model priors

b0~dnorm(0,.0001) #intercept

b1~dnorm(0,.0001) #riparian vegetation

b2~dnorm(0,.0001) #pH

#detection model priors

alpha.a0~dnorm(0,.0001) #intercept mean

tau.a0~dgamma(.001,.001) #intercept variance

a1~dnorm(0,.0001) #sunshine

a4~dnorm(0,.0001) #water temperature

#likelihood

#where y is the number scoops containing larvae at a particular site (known)

#where n is the number of trials at a particular site (known)

#where veg & ph are site level explanatory variables (known)

#where sunny & temp are explanatory variables for each dip (known)

#where p is detection probability (unknown)

#where z is the true state of occupancy (unknown)

for(i in 1:n.sites){

logit(psi[i])<-b0+b1*(veg[i]-mean(veg))+b2*(ph[i]-mean(ph)) #relate veg & ph to true state

z[i] ~ dbern(psi[i])

logit(p[i])<-alpha.a0 +a1*sunny.site[i] + a4*temp.site[i] #take site level data to calculate detection at each site based on the detection function (below)

tmp[i]<-z[i]*p[i] #multiply true state by detection probability

y.ano[i]~dbin(tmp[i],n[i]) #relate to observed state

##get WAIC output for R function

w[i]<-dbin(y.ano[i],tmp[i],n[i])

log.w[i]<-log(w[i])

} #close loop i

#then model detection based on individual samples

#added random intercept for repeated group sampling

for(j in 1:n.all){

logit(psi.p[j])<- a0[group.ano.ind[j]] + a1*sunny[j] + a4*temp[j]

y.p.ano[j]~dbern(psi.p[j])

}

#for random intercept to allow for repeated samplings at same sites

for(k in 1:26){

a0[k]~dnorm(alpha.a0,tau.a0)

}

} #close model
