# Supplementary material for: The importance of accounting for larval detectability in mosquito habitat-association studies
Source: Malar J. 2016 May 4;15:253. doi: 10.1186/s12936-016-1308-4 (PMC4855760; doi:10.1186/s12936-016-1308-4)
Supplement: Supplementary file 3 — 10.1186/s12936-016-1308-4 Table of outputs for the GLMM modelling using multi-model inference. [file 12936_2016_1308_MOESM3_ESM.docx]

**Additional file 3: Tables**

Outputs for the GLMM modeling using multi-model inference (MuMIn). Candidate models ranked using AIC.

(a) presence-absence GLMM model

AIC-ranked candidate model output for presence-absence logistic regression model (logit-link), with site as a random effect. Models are ranked based on inclusion of the following fixed effects

1. algae
2. riparian vegetation
3. depth
4. pH
5. water temperature
6. sunshine on the water surface

# model df logLik AICc Delta Weight

# 234 5 -18.64 48.86 0.00 0.14

# 23 4 -20.07 49.16 0.30 0.12

# 24 4 -20.10 49.23 0.37 0.12

# 1234 6 -18.04 50.35 1.49 0.07

# 12346 7 -16.74 50.59 1.73 0.06

# 245 5 -19.73 51.03 2.17 0.05

# 124 5 -19.76 51.09 2.23 0.05

# 2345 6 -18.44 51.16 2.30 0.05

# 236 5 -19.81 51.20 2.34 0.04

# 123 5 -19.86 51.29 2.43 0.04

# 235 5 -19.87 51.33 2.46 0.04

# 2346 6 -18.56 51.39 2.53 0.04

# 246 5 -20.09 51.75 2.89 0.03

# 12345 7 -17.88 52.87 4.01 0.02

# 1245 6 -19.42 53.12 4.26 0.02

# 1236 6 -19.60 53.47 4.61 0.01

# 2456 6 -19.64 53.56 4.70 0.01

# 1235 6 -19.68 53.63 4.77 0.01

# 2356 6 -19.71 53.70 4.84 0.01

# 1246 6 -19.74 53.75 4.89 0.01

# 23456 7 -18.42 53.96 5.09 0.01

# 2 3 -24.33 55.25 6.39 0.01

# 25 4 -23.13 55.28 6.42 0.01

# 12456 7 -19.33 55.77 6.91 0.00

# 123456 8 -17.85 55.81 6.95 0.00

# 12356 7 -19.51 56.13 7.27 0.00

# 26 4 -24.29 57.61 8.75 0.00

# 12 4 -24.32 57.66 8.80 0.00

# 256 5 -23.12 57.83 8.97 0.00

# 125 5 -23.13 57.83 8.97 0.00

# 126 5 -24.28 60.14 11.28 0.00

# 1256 6 -23.12 60.52 11.66 0.00

# (Null) 2 -29.93 64.16 15.30 0.00

# 4 3 -28.81 64.22 15.36 0.00

# 5 3 -29.01 64.62 15.76 0.00

# 3 3 -29.18 64.95 16.09 0.00

# 1 3 -29.52 65.64 16.78 0.00

# 45 4 -28.41 65.84 16.98 0.00

# 46 4 -28.52 66.07 17.21 0.00

# 6 3 -29.77 66.13 17.27 0.00

# 56 4 -28.59 66.20 17.34 0.00

# 14 4 -28.60 66.22 17.36 0.00

# 34 4 -28.70 66.43 17.57 0.00

# 15 4 -28.71 66.44 17.58 0.00

# 35 4 -28.73 66.48 17.62 0.00

# 13 4 -28.94 66.90 18.04 0.00

# 36 4 -29.05 67.12 18.26 0.00

# 456 5 -27.91 67.40 18.54 0.00

# 16 4 -29.39 67.80 18.94 0.00

# 145 5 -28.22 68.02 19.16 0.00

# 146 5 -28.34 68.25 19.39 0.00

# 156 5 -28.36 68.29 19.43 0.00

# 345 5 -28.38 68.34 19.48 0.00

# 356 5 -28.41 68.39 19.53 0.00

# 346 5 -28.47 68.53 19.66 0.00

# 135 5 -28.51 68.60 19.74 0.00

# 134 5 -28.52 68.63 19.77 0.00

# 136 5 -28.83 69.23 20.37 0.00

# 1456 6 -27.78 69.83 20.97 0.00

# 3456 6 -27.91 70.09 21.23 0.00

# 1345 6 -28.21 70.69 21.83 0.00

# 1356 6 -28.23 70.72 21.86 0.00

# 1346 6 -28.31 70.88 22.02 0.00

# 13456 7 -27.77 72.66 23.80 0.00

(b) success-trial GLMM model

AIC-ranked candidate model output for success-trial binomial GLMM regression model (logit-link), with site as a random effect. Models are ranked based on inclusion of the following fixed effects

1. algae
2. riparian vegetation
3. depth
4. pH
5. water temperature
6. sunshine on the water surface

model df logLik AICc Delta Weight

# 236 5 -70.61 152.79 0.00 0.17

# 2356 6 -69.34 152.95 0.16 0.16

# 12346 7 -68.03 153.17 0.38 0.14

# 1236 6 -69.47 153.22 0.43 0.14

# 12356 7 -68.21 153.52 0.73 0.12

# 2346 6 -69.93 154.13 1.34 0.09

# 123456 8 -67.17 154.46 1.67 0.08

# 23456 7 -68.96 155.03 2.24 0.06

# 1246 6 -71.31 156.88 4.09 0.02

# 12456 7 -70.40 157.91 5.12 0.01

# 246 5 -74.91 161.40 8.61 0.00

# 2456 6 -73.70 161.67 8.88 0.00

# 1256 6 -73.87 162.01 9.22 0.00

# 126 5 -75.33 162.24 9.45 0.00

# 256 5 -76.05 163.68 10.89 0.00

# 26 4 -77.79 164.61 11.82 0.00

# 356 5 -80.33 172.24 19.45 0.00

# 1356 6 -79.26 172.80 20.01 0.00

# 136 5 -80.77 173.11 20.32 0.00

# 36 4 -82.08 173.18 20.39 0.00

# 1346 6 -80.21 174.69 21.90 0.00

# 146 5 -81.56 174.70 21.91 0.00

# 3456 6 -80.26 174.80 22.01 0.00

# 13456 7 -78.85 174.82 22.03 0.00

# 1456 6 -80.50 175.26 22.47 0.00

# 346 5 -81.96 175.49 22.70 0.00

# 1245 6 -80.62 175.52 22.73 0.00

# 16 4 -83.32 175.66 22.87 0.00

# 156 5 -82.04 175.66 22.87 0.00

# 245 5 -82.61 176.81 24.02 0.00

# 124 5 -82.67 176.92 24.13 0.00

# 56 4 -83.98 176.98 24.19 0.00

# 12345 7 -80.04 177.19 24.40 0.00

# 2345 6 -81.62 177.50 24.71 0.00

# 6 3 -85.55 177.70 24.91 0.00

# 456 5 -83.11 177.80 25.01 0.00

# 1234 6 -81.85 177.98 25.19 0.00

# 46 4 -84.52 178.07 25.28 0.00

# 234 5 -83.39 178.37 25.58 0.00

# 24 4 -84.69 178.41 25.62 0.00

# 235 5 -83.90 179.37 26.58 0.00

# 1235 6 -83.20 180.68 27.89 0.00

# 23 4 -86.42 181.86 29.07 0.00

# 25 4 -86.71 182.44 29.65 0.00

# 125 5 -85.64 182.85 30.06 0.00

# 123 5 -85.84 183.26 30.47 0.00

# 2 3 -89.96 186.51 33.72 0.00

# 12 4 -88.85 186.73 33.94 0.00

# 145 5 -91.43 194.44 41.65 0.00

# 45 4 -92.73 194.49 41.69 0.00

# 5 3 -94.18 194.95 42.16 0.00

# 15 4 -93.28 195.58 42.79 0.00

# 35 4 -93.65 196.32 43.53 0.00

# 14 4 -93.78 196.58 43.79 0.00

# 4 3 -95.13 196.86 44.07 0.00

# 345 5 -92.65 196.89 44.10 0.00

# 1345 6 -91.43 197.13 44.34 0.00

# 135 5 -92.93 197.44 44.65 0.00

# (Null) 2 -97.09 198.47 45.68 0.00

# 1 3 -96.14 198.88 46.09 0.00

# 134 5 -93.77 199.12 46.33 0.00

# 34 4 -95.08 199.19 46.40 0.00

# 3 3 -96.54 199.68 46.89 0.00

# 13 4 -95.82 200.68 47.88 0.00
