# Supplementary material for: The importance of accounting for larval detectability in mosquito habitat-association studies
Source: Malar J. 2016 May 4;15:253. doi: 10.1186/s12936-016-1308-4 (PMC4855760; doi:10.1186/s12936-016-1308-4)
Supplement: Supplementary file 4 — 10.1186/s12936-016-1308-4 Table of coefficient estimates for the presence-detection mixture model when terms with effect probabilities of <90 % were removed. [file 12936_2016_1308_MOESM4_ESM.docx]

**Additional file 4: Tables**

Mean ± standard deviation of the posterior distribution (with 95% credible intervals) for coefficients fitted in the presence-detection mixture model when terms with effect probabilities of <90% in the full model (Table 2) were sequentially dropped from the model. For each coefficient the proportion of the posterior distribution that lies above (or below) zero is also shown as the ‘effect probability’: this is the probability that the effect of the parameter on larval presence or detection is in the direction specified by the sign in front of the coefficient (i.e. complete certainty = 1; complete uncertainty = 0.5). See Table 1 for definition of parameters.

|  | Presence | |  |
| --- | --- | --- | --- |
|  |  |  |  |
| Parameter | Posterior distribution | Effect probability |  |
|  |  |  |  |
| Intercept | 0.49 ± 0.60 (-0.48, 1.82) | - |  |
| Vegetation | -0.15 ± 0.06 (-0.29, -0.07) | 1 |  |
| Depth | -0.45 ± 0.28 (-1.07, -0.01) | 0.980 |  |
| pH | 1.59 ± 1.07 (-0.33, 3.89) | 0.941 |  |
| Sunshine | - | - |  |
| Temperature | - | - |  |
| Algae | - | - |  |

|  |  | Detection | |
| --- | --- | --- | --- |
|  |  |  |  |
| Parameter |  | Posterior distribution | Effect probability |
|  |  |  |  |
| Intercept |  | -0.12 ± 0.34 (-0.80, 0.54) | - |
| Vegetation |  | - | - |
| Depth |  | - | - |
| pH |  | - | - |
| Sunshine |  | 1.17 ± 0.35 (0.48, 1.87) | 0.999 |
| Temperature |  | 0.18 ± 0.06 (0.07, 0.29) | 0.999 |
| Algae |  | 0.652± 0.40 (-0.24, 1.31) | 0.907 |
