# Supplementary material for: The importance of accounting for larval detectability in mosquito habitat-association studies
Source: Malar J. 2016 May 4;15:253. doi: 10.1186/s12936-016-1308-4 (PMC4855760; doi:10.1186/s12936-016-1308-4)
Supplement: Supplementary file 5 — 10.1186/s12936-016-1308-4 Figure showing the relationship between pH and larval presence. [file 12936_2016_1308_MOESM5_ESM.docx]

**Additional file 5: Figure**

The relationship between water pH and the probability of larvae being present in one of the water bodies examined in this study. The line shows the mean and the dashed lines the 95% credible intervals from the posterior distribution of the model prediction.
