# Supplementary material for: The importance of accounting for larval detectability in mosquito habitat-association studies
Source: Malar J. 2016 May 4;15:253. doi: 10.1186/s12936-016-1308-4 (PMC4855760; doi:10.1186/s12936-016-1308-4)
Supplement: Supplementary file 6 — 10.1186/s12936-016-1308-4 Table of cumulative probabilities of detecting larvae based on the number of dips, sunshine and water temperature. [file 12936_2016_1308_MOESM6_ESM.docx]

**Additional file 6: Tables**

(a) The mean cumulative probability (with lower and upper values of the 95% credible intervals) of detecting larvae based on the number of dip samples taken at a site based on conditions of sunshine and very warm water temperatures (~34 degrees C)

|  |  |  | Ideal conditions | |
| --- | --- | --- | --- | --- |
| Number of dips | | lower | Mean | upper |
| 1 |  | 0.824248851 | 0.918523557 | 0.969254617 |
| 2 |  | 0.969111534 | 0.993361589 | 0.999054721 |
| 3 |  | 0.994571316 | 0.999459126 | 0.999970937 |
| 4 |  | 0.999045903 | 0.999955932 | 0.999999106 |
| 5 |  | 0.999832316 | 0.999996409 | 0.999999973 |
| 6 |  | 0.999970529 | 0.999999707 | 0.999999999 |
| 7 |  | 0.999994821 | 0.999999976 | 1 |
| 8 |  | 0.99999909 | 0.999999998 | 1 |
| 9 |  | 0.99999984 | 1 | 1 |
| 10 |  | 0.999999972 | 1 | 1 |
| 11 |  | 0.999999995 | 1 | 1 |
| 12 |  | 0.999999999 | 1 | 1 |
| 13 |  | 1 | 1 | 1 |
| 14 |  | 1 | 1 | 1 |
| 15 |  | 1 | 1 | 1 |
| 16 |  | 1 | 1 | 1 |
| 17 |  | 1 | 1 | 1 |
| 18 |  | 1 | 1 | 1 |
| 19 |  | 1 | 1 | 1 |
| 20 |  | 1 | 1 | 1 |
| 21 |  | 1 | 1 | 1 |
| 22 |  | 1 | 1 | 1 |
| 23 |  | 1 | 1 | 1 |
| 24 |  | 1 | 1 | 1 |
| 25 |  | 1 | 1 | 1 |
| 26 |  | 1 | 1 | 1 |
| 27 |  | 1 | 1 | 1 |
| 28 |  | 1 | 1 | 1 |
| 29 |  | 1 | 1 | 1 |
| 30 |  | 1 | 1 | 1 |

(b) The mean cumulative probability (with lower and upper values of the 95% credible intervals) of detecting larvae based on the number of dip samples taken at a site based on conditions of cloudy weather and cold water temperatures (~20 degrees C)

|  |  |  | Poorest conditions | |
| --- | --- | --- | --- | --- |
| Number of dips | | lower | Mean | upper |
| 1 |  | 0.097717113 | 0.238829323 | 0.462275967 |
| 2 |  | 0.185885592 | 0.4206192 | 0.710852862 |
| 3 |  | 0.265438501 | 0.558992324 | 0.844518632 |
| 4 |  | 0.33721773 | 0.664317889 | 0.91639393 |
| 5 |  | 0.4019829 | 0.74448862 | 0.955043005 |
| 6 |  | 0.460419404 | 0.80551223 | 0.975825543 |
| 7 |  | 0.513145662 | 0.851961613 | 0.987000813 |
| 8 |  | 0.560719663 | 0.88731752 | 0.993010024 |
| 9 |  | 0.603644869 | 0.914229401 | 0.996241322 |
| 10 |  | 0.642375548 | 0.934713935 | 0.997978868 |
| 11 |  | 0.677321577 | 0.950306162 | 0.998913189 |
| 12 |  | 0.708852781 | 0.962174507 | 0.999415595 |
| 13 |  | 0.737302847 | 0.971208344 | 0.999685752 |
| 14 |  | 0.762972854 | 0.978084636 | 0.999831021 |
| 15 |  | 0.786134462 | 0.983318667 | 0.999909136 |
| 16 |  | 0.807032785 | 0.987302659 | 0.99995114 |
| 17 |  | 0.825888984 | 0.990335156 | 0.999973727 |
| 18 |  | 0.84290261 | 0.992643404 | 0.999985872 |
| 19 |  | 0.858253714 | 0.994400375 | 0.999992403 |
| 20 |  | 0.872104751 | 0.99573773 | 0.999995915 |
| 21 |  | 0.884602306 | 0.996755685 | 0.999997803 |
| 22 |  | 0.895878635 | 0.997530522 | 0.999998819 |
| 23 |  | 0.906053074 | 0.998120306 | 0.999999365 |
| 24 |  | 0.915233297 | 0.998569232 | 0.999999658 |
| 25 |  | 0.923516454 | 0.998910941 | 0.999999816 |
| 26 |  | 0.930990206 | 0.999171041 | 0.999999901 |
| 27 |  | 0.937733643 | 0.99936902 | 0.999999947 |
| 28 |  | 0.943818132 | 0.999519717 | 0.999999971 |
| 29 |  | 0.949308062 | 0.999634423 | 0.999999985 |
| 30 |  | 0.954261532 | 0.999721733 | 0.999999992 |
